# The Complete Mitochondrial Genomes of Three Bristletails (Insecta: Archaeognatha): The Paraphyly of Machilidae and Insights into Archaeognathan Phylogeny

**DOI:** 10.1371/journal.pone.0117669

**Published:** 2015-01-30

**Authors:** Yue Ma, Kun He, Panpan Yu, Danna Yu, Xuefang Cheng, Jiayong Zhang

**Affiliations:** 1 Institute of Ecology, Zhejiang Normal University, Jinhua, Zhejiang Province, China; 2 College of Chemistry and Life Science, Zhejiang Normal University, Jinhua, Zhejiang Province, China; Saint Mary’s University, CANADA

## Abstract

The order Archaeognatha was an ancient group of Hexapoda and was considered as the most primitive of living insects. Two extant families (Meinertellidae and Machilidae) consisted of approximately 500 species. This study determined 3 complete mitochondrial genomes and 2 nearly complete mitochondrial genome sequences of the bristletail. The size of the 5 mitochondrial genome sequences of bristletail were relatively modest, containing 13 protein-coding genes (PCGs), 2 ribosomal RNA (rRNA) genes, 22 transfer RNA (tRNA) genes and one control region. The gene orders were identical to that of *Drosophila yakuba* and most bristletail species suggesting a conserved genome evolution within the Archaeognatha. In order to estimate archaeognathan evolutionary relationships, phylogenetic analyses were conducted using concatenated nucleotide sequences of 13 protein-coding genes, with four different computational algorithms (NJ, MP, ML and BI). Based on the results, the monophyly of the family Machilidae was challenged by both datasets (W12 and G12 datasets). The relationships among archaeognathan subfamilies seemed to be tangled and the subfamily Machilinae was also believed to be a paraphyletic group in our study.

## Introduction

Bristletails (Insecta: Archaeognatha) were primarily wingless insects inhabiting seashore or inland habitats all around the world, and they typically lived under rocks, logs, and the loose bark of standing trees. The most conspicuous features of these insects were the large compound eyes that meet dorsally, the well developed ocelli and the ability of these insects to spring into the air by a sudden flexure of the body [1–2]. Except for a rare few, bristletails were nocturnal and typically hided in crevices during the day. Archaeognatha was an ancient insect order with modest species diversity (about 500 species in two extant families: Machilidae and Meinertellidae) and the family Machilidae was divided into three subfamilies: Petrobiellinae, Petrobiinae and Machilinae [3]. The Meinertellidae occured predominantly in the Southern Hemisphere [4]. The Machilidae, considered by many authors to possess more primitive features than the Meinertellidae [3], were mostly found in the Northern Hemisphere. Relatively few cladistics analyses had addressed relationships among the three subfamilies of Machilidae and most hypotheses on infraordinal relationships were based on morphological evidence [5–6]. Among the three subfamilies, Machilinae was believed to occupy a series of primitive morphological characteristics, while the species of Petrobiinae and Petrobiellinae were more derived compared with Machilinae species based on the apomorphies such as the absence of scales of antennae and differences of penis feature and abdominal styli. Consequently, a tentative phylogenetic relationship (Machilinae + (Petrobiinae + Petrobiellinae)) was proposed [3].

True insects contained about two dozen orders of winged insects (Pterygota) and two orders of primitively wingless insects, Archaeognatha and Zygentoma. Zygentoma was the putative sister group of the pterygote insects; these two together formed the clade Dicondylia, with Archaeognatha as its sister group. Non-insect hexapods (the so-called Entognatha) comprised the orders Protura, Collembola, and Diplura. The phylogenetic relationships between and within the different orders which composed the primitively wingless hexapods, the so-called apterygote insects, and also their relations with the pterygote hexapods, had been subject to controversial assumptions [7–10]. Even though basal hexapods had modest species diversity, interesting relict distributions, and they clearly were phylogenetically significant, the phylogenetic work on these orders had so far been surprisingly preliminary [11–14].

The insect mitochondrial genome was a compact circular molecule typically 15–18 kb in size and typically encoded for 13 proteins, 22 tRNAs and 2 rRNAs. In addition to the genes, there was a large non-coding region termed the control region as it regulated the transcription and replication of the mtDNA [15–16]. Mitochondrial genome sequences were becoming increasingly important for comprehensive evolutionary studies. MtDNA had been widely regarded as the molecular marker for the phylogenetic analysis in metazoans because of its abundance in animal tissues, the small genome size, faster rate of evolution, low or absence of sequence recombination, and evolutionary conserved gene products [17–18]. Mitochondrial genome sequences were not only more informative than shorter sequences of individual genes, but also provided a set of genomic characters, such as the relative position of different genes, RNA secondary structures and modes of control of replication and transcription [19–22]. To date, over 100 publications reported insect phylogenies built using mitochondrial genome data [23].

Here we described the complete mitochondrial genomes of 3 bristletails, *Petrobiellus bannaensis*, *Allopsontus halawuensis*, *Allopsontus helanensis*, and 2 nearly complete mitochondrial genome sequences, *Pedetontinus luanchuanensis* and *Petrobiellus puerensis*, all of which belonged to the family Machilidae. *P*. *bannaensis* and *P*. *puerensis* were members of the subfamily Petrobiellinae, and *P*. *luanchuanensis* belonged to the subfamily Petrobiinae, while the other two bristletails were in the Machilinae subfamily. The mitochondrial genome sequences were compared with those of other bristletails available in the GenBank database (*Pedetontus silvestrii*, *Petrobius brevistylis*, *Trigoniophthalmus alternatus*, *Songmachilis xinxiangensis* and *Nesomachilis australica*).The protein-coding gene sequences were used to construct datasets for phylogenetic analysis of Archaeognatha. In addition, the genomic sequences were also employed for the inference of the phylogenetic relationships among basal lineages of Hexapoda.

## Material and Methods

### Ethics statement

No specific permits were required for the insect collected for this study. The field studies did not involve endangered or protected species. The species in the order Archaeognatha were common small insects living under rocks or dead leaves and were not included in the ‘‘List of Protected Animals in China”.

### Specimen collection

Specimens of *P*. *bannaensis* were collected from Xishuangbanna of Yunnan province (E101° 14′, N21° 55′), *P*. *puerensis* from Puer of Yunnan province (E101° 2′, N23° 5′), *A*. *helanensis* (E105° 51′, N38° 58′) and *A*. *halawuensis* (E106° 8′, N39° 1′) from Ho-lan Mountains of Gansu province and *P*. *luanchuanensis* (E111° 45′, N33° 42′) from Luanchuan of Henan province. Voucher specimens were deposited in College of Chemistry and Life Sciences, Zhejiang Normal University, Zhejiang, China. All specimens were preserved in 100% ethanol in field. After being transported to the laboratory, they were stored at −70°C until used for DNA extraction. All the five species were identified by morphological characteristics.

### DNA extraction, PCR amplification and sequencing

Total genomic DNA from each individual was extracted using the QIAGEN DNeasy Blood& Tissue Kit following the manufacturer’s protocol. DNA samples were stored at −20°C until further use. Based on alignments and comparisons of complete mitochondrial sequences of other bristletails and our previous researches [24], ten pairs of universal primers were designed for amplification of the mitochondrial genomes (S1 Table). All PCRs were performed using a Bio-RAD MJ Mini Personal Thermal Cycler. Based on the sequence information got from earlier PCR by universal primers, several pairs of specific primers were designed to obtain the whole mitochondrial genome. PCR reactions were carried out in a 50 μL reaction volume consisting of 32.75 μL sterile deionized water, 5.0 μL 10×PCR Buffer (Mg^2+^Free), 5.0 μL MgCl_2_ (25 mM), 4.0 μL dNTP Mixture (2.5 mM each), 1.0 μL DNA template, 1.0 μL each primer (10 ppm), 0.25 μL Takara Taq DNA polymerase (5 U/μL) with the following conditions: after an initial denaturation at 94°C for 4 min, then 94°C for 40 s (denaturation), 46–62°C for 50 s (annealing), 72°C for 1–5 min (extension) for 35 cyclers, followed by 72°C for 10 min (final extension). Each amplicon (5μL) was examined by agarose gel electrophoresis to validate amplification efficiency. After being purified by Axygen agarose-out kit, all the amplicons were sent to Sangon Biotech Company (Shanghai, China) for sequencing from both directions by using the primers used in the PCR amplification.

### Sequence analysis and annotation

All of the typical protein-coding genes were identified by BLAST searches of NCBI database. Sequences were checked and assembled using the programs Seqman (DNASTAR, Inc.) and then adjusted manually. PCGs and rRNA genes were identified based on sequences similarity with published bristletail mitochondrial genome sequences from public database (e.g., GenBank). The base composition, codon usage, and open-reading frames were analyzed using program Mega 5.0 [25]. Translation initiation and translation termination codons were identified based on comparison with the mitochondrial genomes of other known bristletails. The overlapping regions and intergenic spacers between genes were counted manually. The tRNA genes were identified by their cloverleaf secondary structure using tRNAscan-SE Search Server v.1.21 [26] with default settings. The tRNA genes which could not be determined by tRNAscan-SE were determined by sequence similarity to tRNAs of other bristletails. Sequence data had been deposited in the NCBI database, and the accession numbers of *Petrobiellus bannaensis*, *Allopsontus halawuensis*, *Allopsontus helanensis*, *Pedetontinus luanchuanensis* and *Petrobiellus puerensis* were KJ754503, KJ754500, KJ754501, KJ754502 and KJ754504, respectively.

### Phylogenetic analyses of Archaeognatha and basal Hexapoda

To elucidate the phylogenetic relationships among basal Hexapoda, 35 sequences of the complete mitochondrial genomes were obtained from GenBank database (Table 1). Among the mitochondrial genomes we selected, there were 5 from the earlier published bristletails [2, 24, 27–29], 3 from another primitive wingless order Zygentoma [30–32], 8 from the Paleoptera order: Ephemeroptera and Odonata [33–36], 4 from the Polyneoptera order: Blattaria [37], Isoptera [38], Mantodea [39] and Orthoptera [40]. To optimize the outgroup selection, we selected 14 entognathan species and 2 Crustacean species as outgroups to construct phylogenetic trees, respectively [28, 30, 41–46]. The nucleotide and putative amino acid regions for each of the 13 mitochondrial protein-coding genes were aligned using Clustal W in the program Mega 5.0 [25]. All the alignments were analyzed with the program Gblock 0.91b using default settings, to select conserved regions of the putative amino acids [47]. A saturation analysis was performed on the first, second and third codon positions of PCGs using DAMBE 4.2.13 [48]. According to the results from saturation analysis, third codon positions were satured, so they were excluded from the final nucleotide alignment. Phylogenetic analyses were conducted using four methods: neighbor-Joining (NJ), maximum parsimony (MP), maximum likelihood (ML) and Bayesian (BI). GTR+I+G model was chosen for the likelihood and Bayesian analyses. The Maximum likelihood analysis (ML) of the nucleotide alignment was performed using PAUP* 4.0b10 with 100 bootstrap replicates [49]. Maximum parsimony analysis (MP) and Neighbor-Joining (NJ) were performed using PAUP* 4.0b10. 1000 bootstrap replicates were generated for the MP analysis with 10 replicates with random taxon order. BI analysis was performed using MrBayes, ver.3.1.2 [50]. For Bayesian Inference, 4 independent Markov chains were simultaneously running for 1000000 generations. Trees were sampled every 100 generations and the first 20% of the generations were discarded as burn-in and the remaining trees were used to calculate Bayesian posterior probabilities.

**Table 1:** List of taxa used in the phylogenetic analyses.

Order	Species	Accession No.	Reference
Crustacea	*Capitulum mitella*	NC_008742	
	*Pollicipes mitella*	AY514042	
Protura	*Sinentomon erythranum*	NC_015982	[41]
Collembola	*Onychiurus orientalis*	NC_006074	[30]
	*Podura aquatica*	NC_006075	[30]
	*Gomphiocephalus hodgsoni*	NC_005438	
	*Cryptopygus antarcticus*	NC_010533	[42]
	*Friesea grisea*	NC_010535	[28]
	*Orchesella villosa*	NC_010534	[28]
	*Bilobella aurantiaca*	NC_011195	
	*Sminthurus viridis*	NC_010536	[28]
	*Tetrodontophora bielanensis*	NC_002735	[43]
	*Friesea grisea*	EU124719	[44]
Diplura	*Japyx solifugus*	NC_007214	[45]
	*Campodea lubbocki*	NC_008234	[46]
	*Campodea fragilis*	NC_008233	[46]
Archaeognatha	*Petrobius brevistylis*	NC_007688	[2]
	*Nesomachilis australica*	NC_006895	[29]
	*Pedetontus silvestrii*	NC_011717	[27]
	*Trigoniophthalmus alternatus*	NC_010532	[28]
	*Songmachilis xinxiangensis*	JX308221	[24]
	*Petrobiellus puerensis*		This study
	*Petrobiellus bannaensis*		This study
	*Allopsonsu halawuensis*		This study
	*Allopsonsus helanensis*		This study
	*Pedetontinus luanchuanensis*		This study
Zygentoma	*Thermobia domestica*	NC_006080	[30]
	*Tricholepidion gertschi*	NC_005437	[30]
	*Atelura formicaria*	NC_011197	[31]
Odonata	*Euphaea formosa*	NC_014493	[34]
	*Davidius lunatus*	NC_012644	[33]
	*Pseudolestes mirabilis*	FJ606784	
	*Orthetrum triangulare melania*	AB126005	[35]
Ephemeroptera	*Siphlonurus immanis*	NC_013822	
	*Ephemera orientalis*	NC_012645	[33]
	*Parafronurus youi*	NC_011359	[32]
	*Habrophlebiodes zijinensis*	GU936203	
Mantodea	*Tamolanica tamolana*	NC_007702	[38]
Isoptera	*Macrotermes barneyi*	NC_018599	[37]
Blattaria	*Blattella bisignata*	NC_018549	[36]
Orthoptera	*Xizicus fascipes*	NC_018765	[39]

We selected *Onychiurus orientalis* from Collembola and *Campodea lubbocki* from Diplura as outgroups to construct the phylogeny of the order Archaeognatha, and for the first time, tried to elucidate the relationships among subfamilies of Archaeognatha using mitochondrial genomes. W12 dataset was constructed based on the nucleotide sequences of all the 13 protein-coding genes, which included all the indels after being aligned; G12 dataset was constructed using the program Gblock 0.91b, this dataset possessed conserved regions of all the 13 protein-coding genes, so all the poorly aligned positions of nucleotide alignment were removed. Then, W12 and G12 datasets were utilized to constructed phylogenetic trees with NJ, MP, ML and BI algorithms, respectively.

## Results and Discussion

### Genome organization

The complete mitochondrial genomes of *P*. *bannaensis*, *A*. *halawuensis*, and *A*. *helanensis* were obtained with the size of 15843 bp, 15532 bp, and 15538 bp, respectively (Table 2). But unfortunately, we were not able to successfully amplify the control region of *P*. *luanchuanensis* and *P*. *puerensis* with the length of amplified sequences of 15196 bp and 14562 bp, respectively. The length of the five mitochondrial genomes were all within the range of typical size for metazoan mt genomes, which was considered to be 14–18 kb [16]. All the 5 mitochondrial genomes contained 13 protein-coding genes (*CO*1–3, *ND*1–6, *ND*4L, *atp*6, *atp*8 and *cob*), a small subunit ribosomal RNA gene (*rrn*S), a large subunit ribosomal RNA gene (*rrn*L), and 22 transfer RNA genes, and 23 of which were transcribed on the majority J-strand and the others mapped to the minority N-strand. The gene orders of the five mitochondrial genomes were congruent with *Drosophila yakuba*, which was regarded as the ancestral metazoan mitochondrial genome [51].

**Table 2:** Annotation of the mitochondrial genomes of *Petrobiellus bannaensis* (Pb), *Allopsontus halawuensis* (Aha) and *Allopsontus helanensis* (Ahe).

Gene/strand	Position			Start/stop codon		
	Pb	Aha	Ahe	Pb	Aha	Ahe
trnI / J	1–69	1–68	1–67			
trnQ / N	83–150	69–138	68–136			
trnM / J	149–217	142–207	140–205			
ND2 / J	227–1246	226–1239	209–1231	ATA/TAA	ATT/TAA	ATC/TAA
trnW / J	1247–1315	1239–1306	1230–1298			
trnC / N	1328–1396	1306–1372	1298–1362			
trnY / N	1407–1471	1373–1440	1363–1430			
Cox1 / J	1506–3005	1433–2972	1423–2962	ATT/TAA	ATT/T	ATT/T
trnL2 / J	3032–3097	2973–3037	2963–3026			
Cox2 / J	3098–3784	3043–3730	3034–3721	ATG/TAA	ATG/T	ATG/T
trnK / J	3783–3854	3731–3802	3722–3792			
trnD / J	3854–3918	3804–3869	3795–3861			
Atp8 / J	3919–4080	3870–4031	3862–4023	ATC/TAA	ATT/TAA	ATT/TAA
Atp6 / J	4074–4751	4028–4702	4017–4691	ATG/TAA	ATA/TAA	ATG/TAA
Cox3 / J	4753–5544	4705–5485	4695–5477	ATG/TAA	ATA/T	ATG/TAA
trnG / J	5549–5612	5503–5567	5482–5547			
ND3 / J	5610–5966	5565–5921	5548–5901	ATA/TAG	ATA/TAA	ATA/TAA
trnA / J	5965–6027	5925–5988	5905–5968			
trnR / J	6033–6099	5996–6060	5971–6037			
trnN / J	6098–6164	6060–6125	6037–6106			
trnS1 / J	6165–6231	6127–6191	6107–6174			
trnE / J	6233–6298	6193–6258	6175–6243			
trnF / N	6308–6373	6261–6326	6244–6308			
ND5 / N	6374–8104	6328–8062	6311–8045	ATT/TAG	ATG/T	ATG/T
trnH / N	8102–8165	8064–8127	8047–8111			
ND4 / N	8171–9499	8133–9482	8115–9455	ATG/TAA	ATG/TAA	ATT/TAA
ND4L / N	9511–9813	9476–9775	9458–9757	ATG/TAA	ATG/TAA	ATG/TAA
trnT / J	9816–9878	9778–9841	9760–9823			
trnP / N	9880–9946	9842–9906	9824–9888			
ND6 / J	9948–10472	9891–10418	9892–10395	ATT/TAA	ATT/TAA	ATT/TAA
CYTP / J	10472–11608	10426–11562	10395–11531	ATG/TAA	ATG/TAA	ATG/TAA
trnS2 / J	11614–11680	11561–11628	11532–11595			
ND1 / N	11702–12634	11648–12586	11616–12536	ATA/TAG	ATT/TAG	GTG/TAA
trnL1 / N	12652–12717	12593–12659	12558–12624			
rrnL / N	12718–14040	12660–13991	12625–13946			
trnV / N	14041–14113	13992–14063	13947–14018			
rrnS / N	14114–14961	14064–14876	14019–14824			
A+T-rich region	14962–15843	14877–15532	14825–15538			

Note: J and N refer to the majority and minority strand, respectively. Position numbers refer to positions on the majority strand.

In the mitochondrial genome of *A*. *halawuensis*, there was a total of 43 bp gene overlap, which was the highest in the 5 mt genomes, and the lowest was *A*. *helanensis* with a total of 20 bp overlap. The 43 bp gene overlap in 9 positions of *A*. *halawuensis* mt genome was ranging in size from 1 to 16 bp and the longest is a 16 bp overlap between *tRNA* ^Pro^ and *ND*6, which was also the longest overlap between individual genes of the 5 mt genomes. Compared to previously published 5 bristletail mt genomes, the gene overlap occurred between *atp*8 and *atp*6 seemed to be common across bristletail mt genomes and this 7 nucleotides “ATGATAA” overlap also existed in other insects [52–53].

In addition to the large non-coding region, several small non-coding intergenic spacers were also found in the 5 mitochondrial genomes. The non-coding regions of mtDNA of *P*. *bannaensis* is 187 bp in total, which is the highest in the 5 mt genomes, and made up of 19 intergenic spacer sequences, ranging in size from 1 bp to 34 bp. There were two relatively large spacers flanked by the protein-coding genes *CO*1 and *ND*1 in the *P*. *bannaensis* mt genome. Interestingly, the intergenic spacers flanked by *ND*1 gene seemed to be a common feature across bristletail mitochondrial genomes. The total nucleotides of intergenic spacers between *tRNA* ^Ser2^, *ND*1 and *tRNA* ^Leu1^ in *P*. *bannaensis*, *A*. *halawuensis*, *A*. *helanensis* and *P*. *puerensis* were 38 bp, 19 bp, 41 bp and 36 bp, respectively. But notably, such feature was not found in the *P*. *luanchuanensis* mt genome, a 8 bp overlap existed between *tRNA* ^Ser2^ and *ND*1 gene.

The nucleotide composition of *P*. *bannaensis*, *A*. *halawuensis*, *A*. *helanensis*, *P*. *luanchuanensis* and *P*. *puerensis* were biased toward A/T nucleotides, which account for 69.7%, 68.1%, 68.9%, 73.2% and 70.7%, respectively, and these values were well within the range detected so far in mitochondrial genomes of bristletail (67.9%- 74.4%).

### Protein-coding genes

13 protein-coding genes were identified in the 5 mitochondrial genomes of *P*. *bannaensis*, *A*. *halawuensis*, *A*. *helanensis*, *P*. *luanchuanensis* and *P*. *puerensis*, with characteristics similar to previously published 5 bristletail mitochondrial genomes. Among the 13 protein-coding genes, 4 genes located on the minority N-strand, and the others were transcribed on the majority J-strand. The commonly used protein-coding gene start codons were ATN, GTG [54] and TTG [55], but there was also reported no typical ATN start codon for *CO*1 gene in mitochondrial genomes of some other species, such as CCG [56], CGA [57], ACC [36], ACG [58], TTA [59] and even a tetranucleotide codon, ATTA, had been proposed in the mitogenome of *Locusta migratoria* [60]. All the 13 protein-coding genes of the 10 bristletail mitochondrial genomes (5 from our work and 5 from NCBI database) started with typical ATN and GTG (Table 2). In the mitochondrial genome of *P*. *bannaensis*, 6 genes shared ATG initiation codon, 3 shared ATT and ATA respectively, and only 1 gene started with ATC. With the similar characteristics in *P*. *bannaensis* mitochondrial genome, most protein-coding genes in bristletail mt genomes started with ATG and ATT. While interestingly, 7 protein-coding genes in *N*. *australica* (from family Meinertellidae) mt genome had a ATA start codon, which was quite different with the other 9 species.

In the *P*. *bannaensis* and *P*. *puerensis* mitochondrial genomes, all the PCGs terminated with the conventional stop codons TAG or TAA. While in terms of the *A*. *halawuensis* mt genome, 4 genes (*CO1*, *CO*2, *CO*3 and *ND*5) had incomplete stop codon T adjacent to downstream tRNA genes. And the incomplete stop codon T was also found in *A*. *helanensis* (*CO*1, *CO*2 and *ND*5) and *P*. *luanchuanensis* (*CO*2, *CO*3 and *ND*5) mitochondrial genomes. The presence of incomplete stop codon was common in metazoan mitochondrial genomes, and these truncated stop codons was presumed to be complete by post-transcriptional polyadenylation [61].

The A+T content of protein-coding gens, excluding stop codons, was 67.3%, 66.6%, 66.9%, 72.2% and 69.6% in *P*. *bannaensis*, *A*. *halawuensis*, *A*. *helanensis*, *P*. *luanchuanensis* and *P*. *puerensis*, respectively. In the 5 mitochondrial genomes, the first, second and third codon positions was calculated separately and the third codon positions seemed to have the highest A+T content (75.7%, 75.5%, 75.8%, 86.9% and 81.3%, respectively), the strongest bias toward T was in the second codon positions of the 5 species (Table 3).

**Table 3:** Nucleotide compositions of *Petrobiellus bannaensis* (Pb), *Allopsontus halawuensis* (Aha) and *Allopsontus helanensis* (Ahe).

feature	A (%)			C (%)			G (%)			T (%)			A+T (%)		
	Pb	Aha	Ahe	Pb	Aha	Ahe	Pb	Aha	Ahe	Pb	Aha	Ahe	Pb	Aha	Ahe
Whole genome	39.0	35.4	36.4	19.5	19.8	19.4	10.9	12.1	11.6	30.7	32.7	32.5	69.7	68.1	68.9
Protein-coding genes	29.5	28.0	27.9	16.7	17.3	17.0	16.0	16.1	16.1	37.8	38.6	39.0	67.3	66.6	66.9
1st codon positions	31.7	28.9	29.3	15.1	16.1	16.2	22.2	23.5	22.6	31.0	31.0	32.0	62.7	59.9	61.3
2nd codon positions	18.1	18.5	18.8	20.3	20.6	20.2	15.8	16.0	15.8	45.9	45.0	45.1	64.0	63.5	63.9
3rd codon positions	38.7	36.5	35.8	14.5	15.2	14.5	9.9	8.9	9.9	37.0	39.0	40.0	75.7	75.5	75.8
tRNA genes	35.5	35.2	37.3	12.2	13.8	12.2	15.5	16.8	15.1	36.9	34.2	35.4	72.4	69.4	72.7
rRNA genes	30.8	33.1	35.1	7.6	9.5	9.3	18.1	19.6	17.4	43.5	37.9	38.2	74.3	71.0	73.3
A+T-rich region	42.7	40.2	43.1	11.9	9.3	11.5	6.2	8.7	5.5	39.0	41.8	39.9	81.7	82.0	83.0

Note: Stop codons were excluded.

### Transfer RNA, Ribosomal RNA genes and A+T rich region

The complete set of 22 tRNAs (one specific for each amino acid and 2 each for leucine and serine) found in typical metazoan mitochondrial genomes were identified in *P*. *bannaensis*, *A*. *halawuensis*, *A*. *helanensis*, *P*. *luanchuanensis* and *P*. *puerensis*. Among these tRNA genes, 14 tRNAs were encoded by the J-strand with the rest by the N-strand. All the tRNA genes could be folded into the typical cloverleaf structure using tRNAscan-SE 1.21 except for tRNA^Ser^ (AGN), in which its dihydrouridine (DHU) stem simply formed a loop, as was often found in other insect mitochondrial genomes. The anticodons of the 5 species were identical to those of the respective tRNAs found in previously published bristletail mitochondrial genomes. Unmatched base pairs had been detected in stems of tRNA secondary structure. Among the 5 mitochondrial genomes, *P*. *bannaensis* possessed 42 unmatched base pairs, consisting of 35 G-U pairs, 2 U-U and 3 U-C mismatches, which was the highest compared to other 5 species. Except for G-U, U-U, U-C pairs and mismatches, the other types of mismatches like A-C and A-A had also been identified. tRNA gene cluster rearrangement occurred between protein-coding genes *ND*3 and *ND*5 had been found in other bristletails. In the mt genomes of *T*. *alternatus* and *P*. *brevistylis*, the tRNA gene orders between *ND*3 and *ND*5 were tRNA^Arg^-tRNA^Asn^-tRNA^Ser1^-tRNA^Glu^-tRNA^Ala^-tRNA^Phe^ and tRNA^Ala^-tRNA^Arg^-tRNA^Asn^-tRNA^Ser1^-tRNA^Glu^-tRNA^Tyr^-tRNA^Phe^, respectively. But in our study, there was no tRNA gene rearrangement that had been detected, and the 5 bristletail species owned the typical insects tRNA gene order within *ND*3 and *ND*5, which was tRNA^Ala^-tRNA^Arg^-tRNA^Asn^-tRNA^Ser1^-tRNA^Glu^-tRNA^Phe^.

The ends of ribosomal RNA genes were impossible to be precisely determined by DNA sequencing alone, so the ends of rRNA genes were assumed to be at the boundaries of flanking genes. As in the 5 mitochondrial genomes, the lrRNA gene was between tRNA^Leu1^ and tRNA^Val^, while the srRNA was flanked by tRNA^Val^ and A+T rich region and both genes were encoded on the minor strand. The length of srRNA in *P*. *bannaensis* mitochondrial genome was 848 bp, which was relatively higher than that of *A*. *halawuensis* (813 bp) and *A*. *helanensis* (806 bp) mt genomes. We could not determine the length of *P*. *luanchuanensis* and *P*. *puerensis* because of our inability to successfully amplify the srRNA gene and A+T rich region. The lengh of lrRNA gene was similar to other bristletail mt genomes.

The largest non-coding region of bristletail mitochondrial genome was the control region, which was located between srRNA and the tRNA^Ile^-tRNA^Gln^-tRNA^Met^ gene cluster. Compared to previously published bristletail mitochondrial genomes, the length of the control region ranged from 538 bp (*N*. *australica*) to 1149 bp (*T*. *alternatus*), and the size of control region in this study were well within this range. The control region length variation was probably due to tandem repeat regions and differences in copy numbers.

### Phylogeny of Archaeognatha and basal Hexapoda

**Fig 1:**
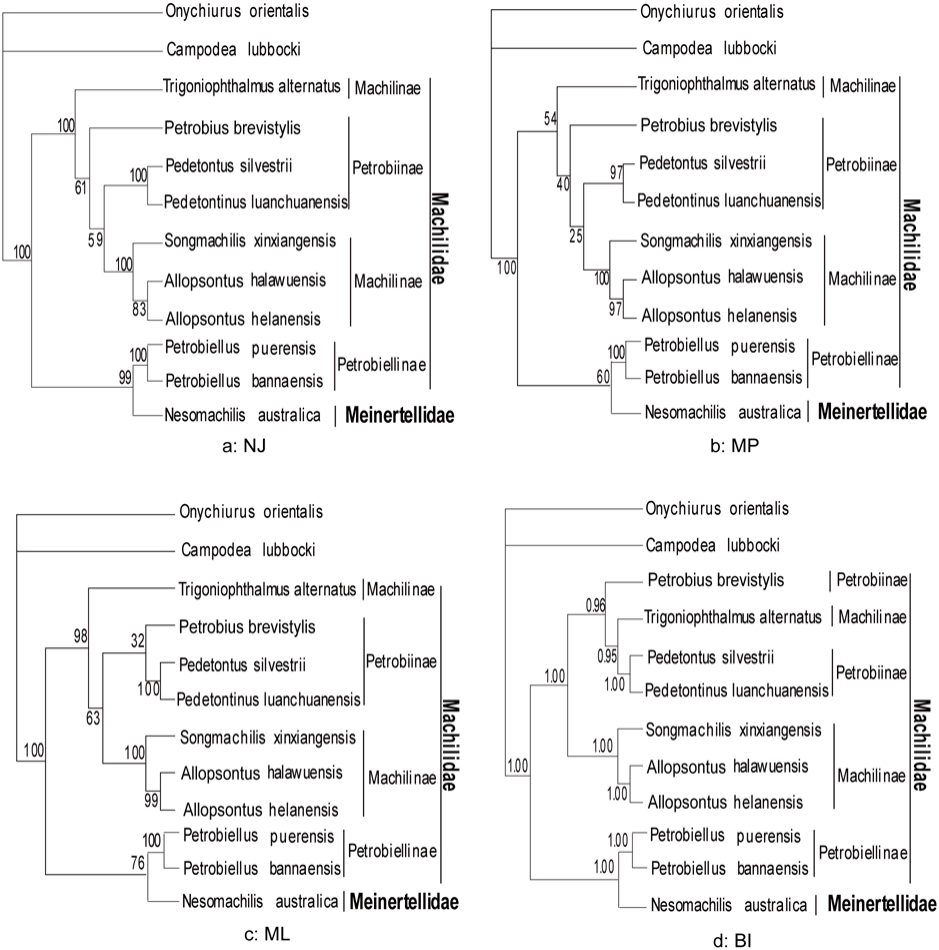
Phylogenetic tree yielded by W12 dataset. All indels after being aligned were reserved in this dataset. Phylogenetic analyses were based on first and second codon positions of 13 protein-coding genes. The tree was rooted with the sequences of two outgroup species: *Onychiurus orientalis* and *Campodea lubbocki*. From W12 dataset, the paraphyly of Machilidae and its subfamily Machilinae was obtained in all algorithms.

**Fig 2:**
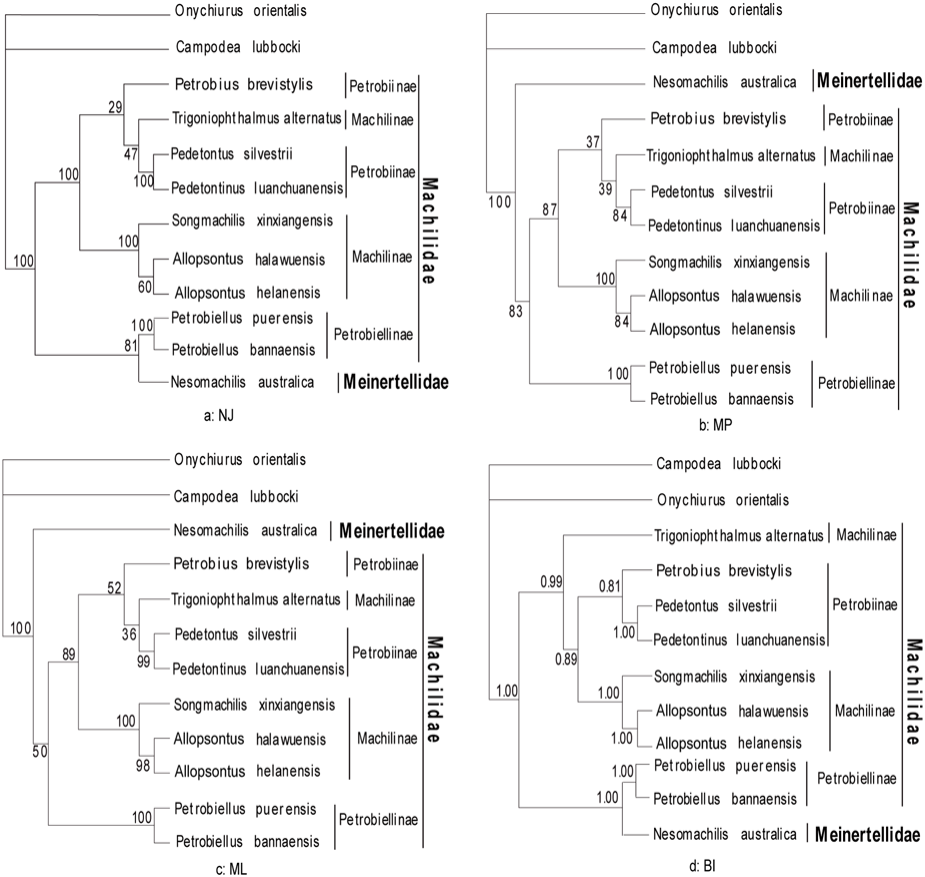
Phylogenetic tree yielded by G12 dataset. All conserved regions after being aligned were retained. Phylogenetic analyses were based on first and second codon positions of 13 protein-coding genes. The tree was rooted with the sequences of two outgroup species: *Onychiurus orientalis* and *Campodea lubbocki*. Almost the same topologies were obtained in G12 dataset compared with W12 dataset.

The Archaeognatha consisted of only 2 extant families and approximately 500 species: Meinertellidae and Machilidae [3]. The Machilidae was considered to possess more primitive features (e.g. relating to ovipositor, penis, parameres, coxal vesicles and tarsomeres) than the Meinertellidae. Kaplin subdivided the family Machilidae into 3 subfamilies: Machilinae, Petrobiinae and Petrobiellinae [5]. And Sturm proposed the monophyly of the 3 subfamilies had to be proved by additional arguments [4]. We performed phylogenetic analysis with nucleotide sequences of 13 mitochondrial protein-coding genes from 10 archaeognathan species and 2 outgroup species (*Onychiurus orientalis* [30] and *Campodea lubbocki* [46]). Four phylogenetic algorithms (NJ, MP, ML and BI) yielded 2 datasets (Fig. 1 and Fig. 2): W12 (13 PCG nucleotide sequences with poorly aligned positions of nucleotide alignment) and G12 (13 PCG nucleotide sequences analyzed by Gblock 0.91b). The 2 datasets showed the following features: firstly, the family Machilidae seemed to be a paraphyletic group, which was not supporting the taxonomic classification by analysis of morphological data. In the W12 dataset, all the analyses support this view, while in the G12 dataset, both NJ and BI analyses yielded the same results. Secondly, the monophyly of the subfamily Machilinae was also challenged by all analyses in this paper. The group consisting of (*S*. *xinxiangensis* + (*A*. *halawuensis* + *A*. *helanensis*)) was strongly supported as being monophyletic across all analyses, but another Machilinae species *T*. *alternatus* was not in this clade. The main morphological characters for the diagnosis of this subfamily by Sturm were plesiomorphies, so he proposed a subdivision or an additional diagnosis was required, and our results suggested the phylogenetic relationships among Machilinae was not congruent with morphological taxonomy. Thirdly, the Machilidae subfamily Petrobiellinae seemed to have a closer relationship with the other family Meinertellidae. *P*. *puerensis* and *P*. *bannaensis* were clustered with the Meinertellidae species *N*. *australica* in most analyses with relatively high nodal supports. This subfamily comprised only one genus *Petrobiellus*, and besides some plesiomorphies, this genus shared an apomorphic feature with the *Petrobius* group: the weak and indistinct separation of the distal mandibular teeth. Sturm suggested that this feature could indicate the *Petrobiellus* group and *Petrobius* were monophyletic or the parallel evolution had taken place [3]. But in our analyses, the genus *Petrobiellus* and *Petrobius* were not in the same clade, interestingly, the 2 *Petrobiellus* species formed a monophyletic clade with the Meinertellidae species *N*. *australica* in most analyses (all analyses in W12 dataset and NJ, BI analyses in G12 dataset). Our result may provide evidence for resolving phylogenetic relationships of Archaeognatha, although the currently limited taxon sampling showed the preliminary nature of this analysis. The unsampled groups should be included in further studies to provide a more comprehensive phylogenetic estimate.

**Fig 3:**
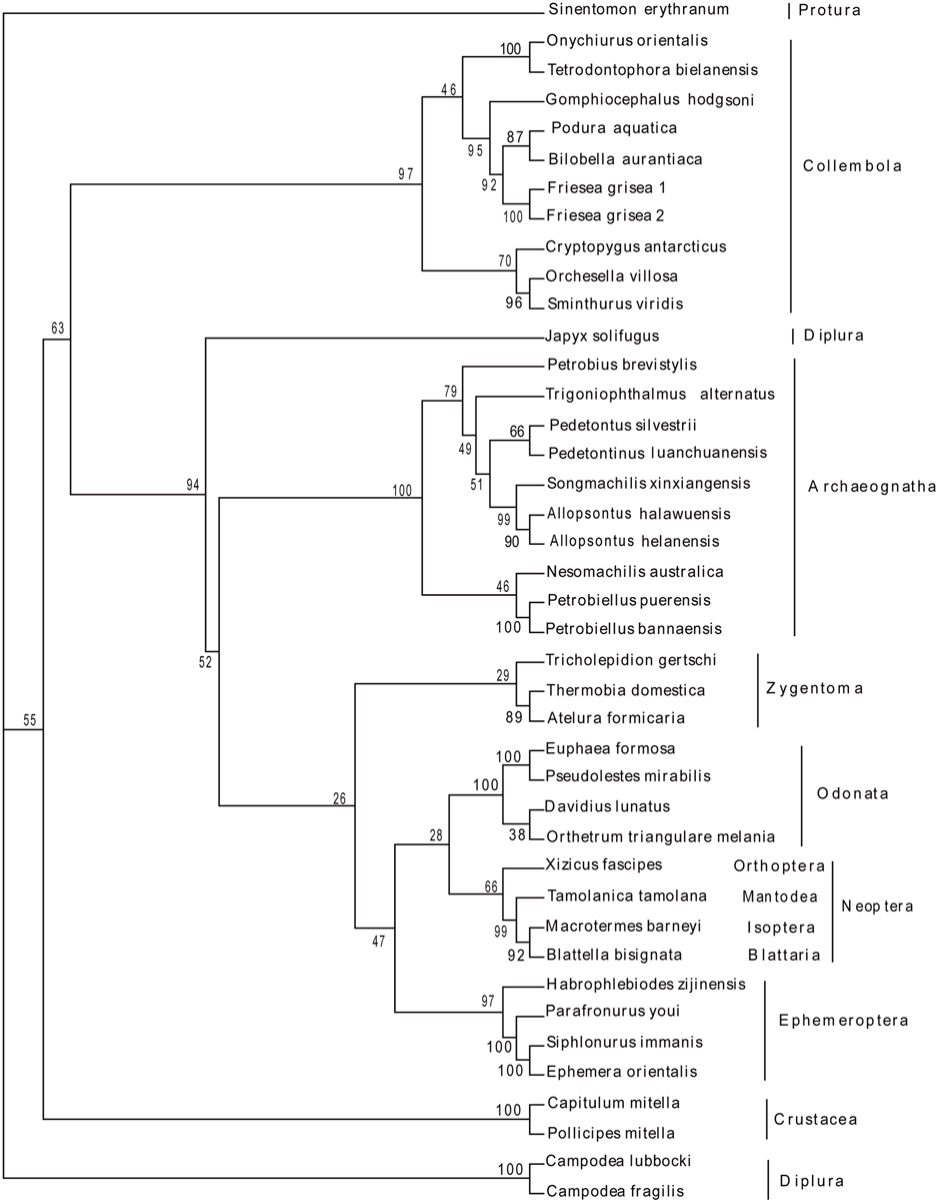
Phylogenetic relationships among basal Hexapods inferred from Maximum parsimony of 13 protein-coding sequences. 1 Protura species, 10 Collembola species and 3 Diplura species were used as the outgroup. Numbers denoted bootstrap values in percentages.

**Fig 4:**
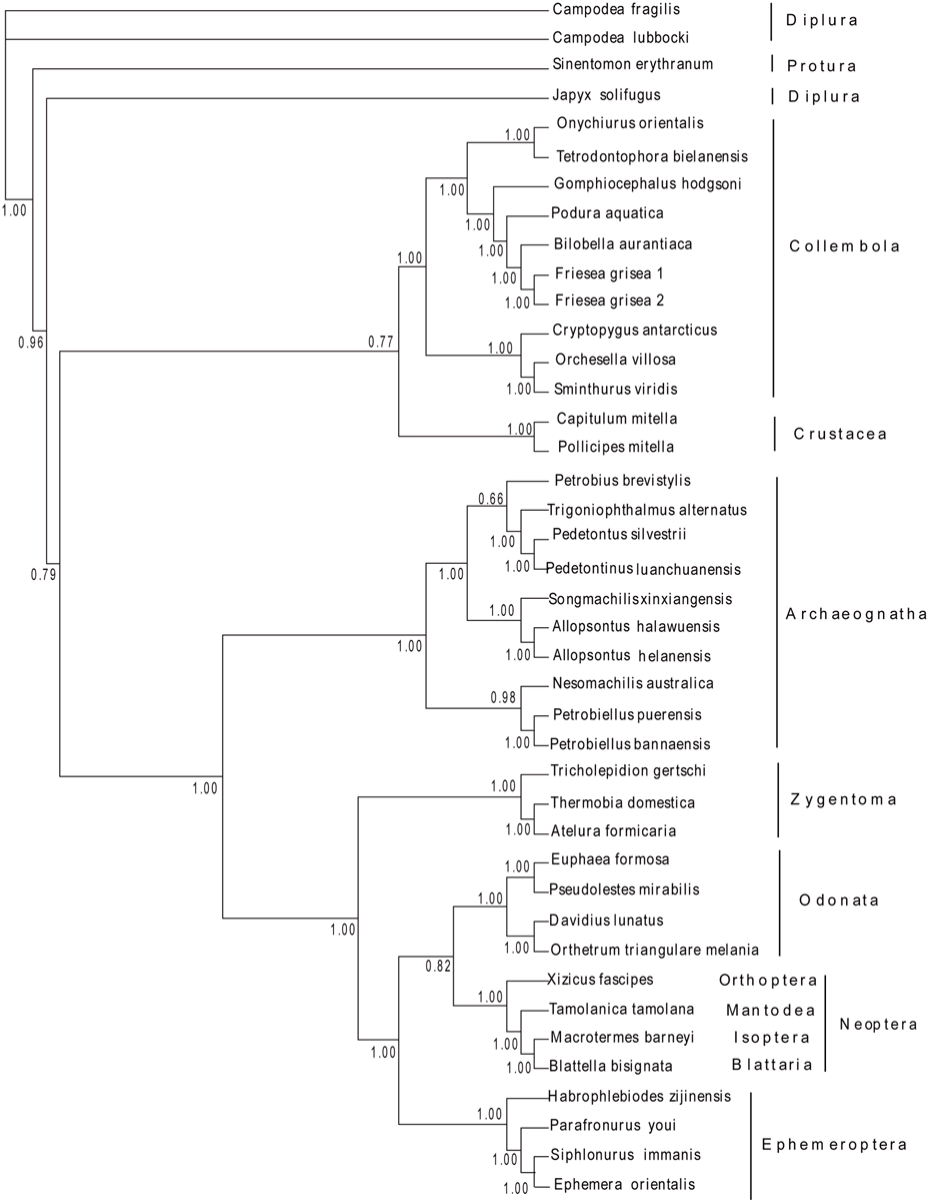
Phylogenetic relationships among basal Hexapods inferred from Bayesian analysis of 13 protein-coding sequences. 1 Protura species, 10 Collembola species and 3 Diplura species were used as the outgroup. Numbers denoted posterior probabilities of nodes.

**Fig 5:**
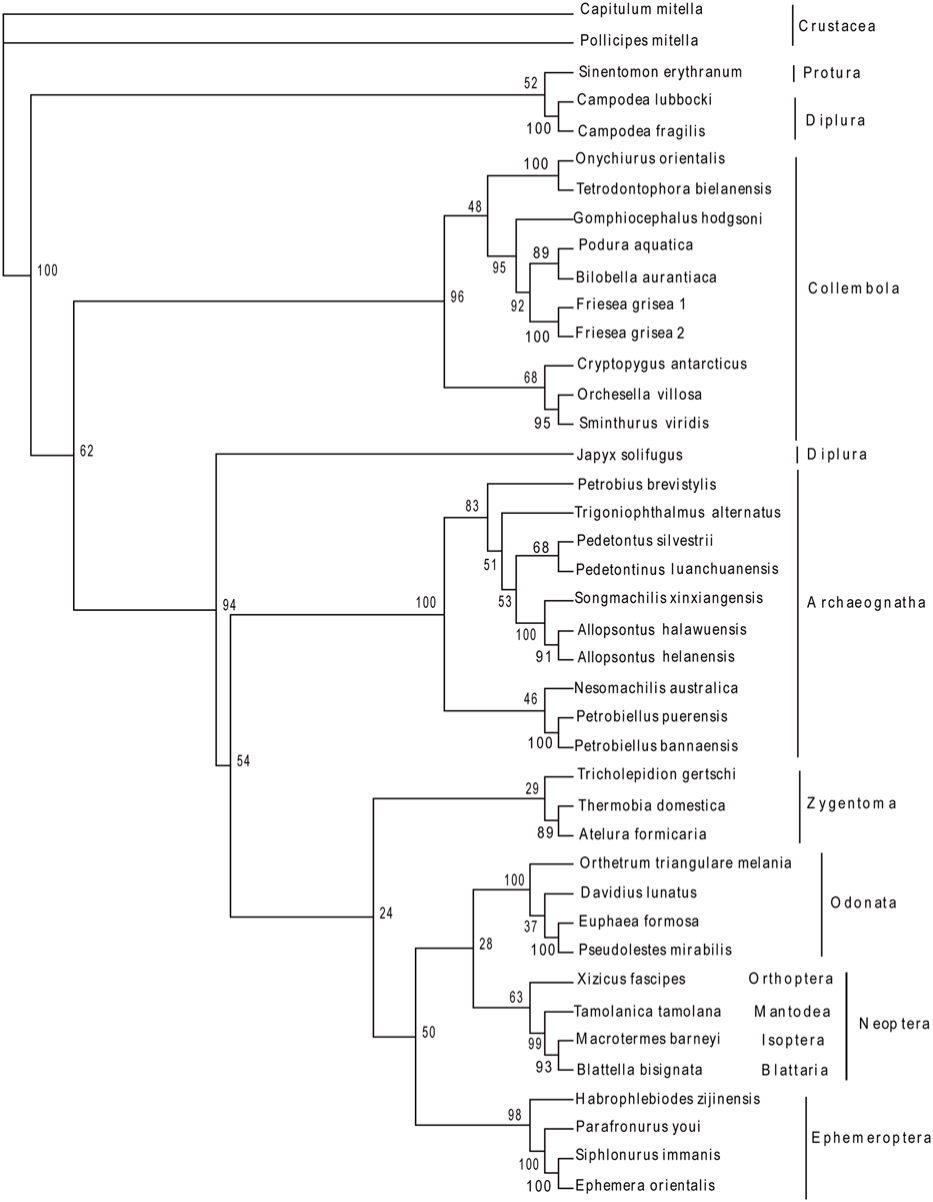
Phylogenetic relationships among basal Hexapods inferred from Maximum parsimony analysis of protein-coding gene sequences. 2 Crustacean species *Capitulum mitella* and *Pollicipes mitella* were used as the outgroup. Numbers denoted bootstrap values in percentages.

**Fig 6:**
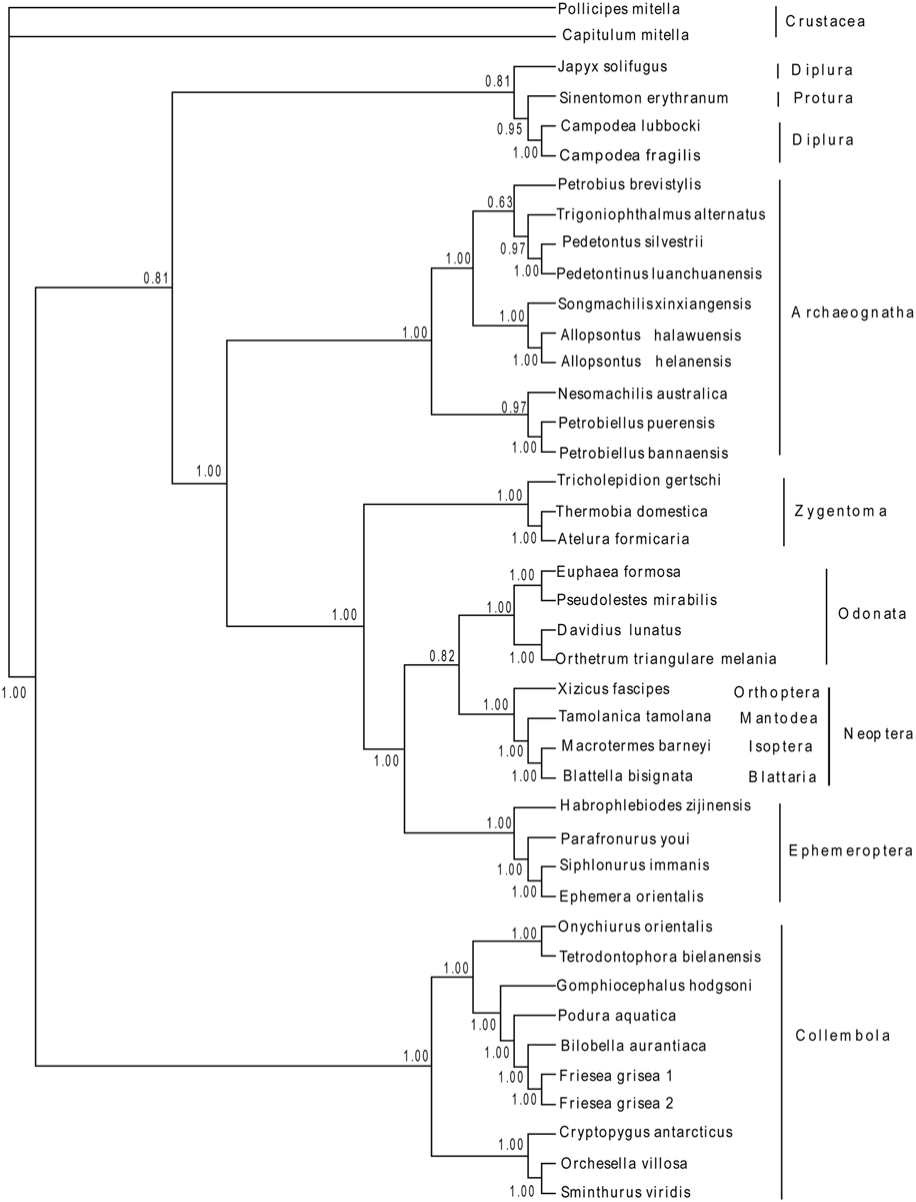
Phylogenetic relationships among basal Hexapods inferred from Bayesian analysis of protein-coding gene sequences. 2 Crustacean species *Capitulum mitella* and *Pollicipes mitella* were used as the outgroup. Numbers denoted posterior probabilities of nodes.

To elucidate the phylogenetic relationships among basal Hexapoda, 35 sequences of the complete mitochondrial genomes were obtained to construct phylogenetic trees using 14 Entognathan species and 2 Crustacean species as outgroups, respectively. Based on the results, the monophyly of Archaeognatha was an undoubted hypothesis strongly supported by both morphological features and molecular data that was also demonstrated in our study based on mitochondrial genomes (Figs. 3, 4, 5 and 6). The topologies showed the similar characteristics with datasets W12 and G12, corroborating the paraphyly of Machilinae and other features demonstrated by those two datasets. The traditional taxon Thysanura was the combination of bristletails and Zygentoma species. However, it had been recognized as an unnatural group in recent years. The topologies showed that silverfish (Zygentoma) were more closely related to the winged insects (pterygota) than to bristletails. Most of our analyses yielded a topology of (Ephemeroptera + (Odonata + Neoptera)), but both NJ analyses yielded a (Odonata + (Ephemeroptera + Neoptera)) clade (S1 Fig. and S3 Fig.), hence, the phylogenetic relationships between the two Paleoptera orders needed a further investigation. The monophyly of Pterygota and Neoptera was also obtained from our analyses. The monophyly of Collembola were verified, but the phylogenetic position of Diplura was challenged by our datasets. All the analyses except for both ML analyses (with relatively low support values) showed that Diplura was a paraphyletic group (S2 Fig. and S4 Fig.). Three dipluran species: *Japyx solifugus*, *Campodea lubbocki* and *Campodea fragilis* were utilized for the phylogenetic analyses. *Japyx solifugus* seemed to have a closer relationship with Archaeognatha and formed a monophyletic group with true insects. The monophyly of Diplura had been questioned because the subgroups Campodeoidea and Japygoidea differed in cerci, sperm morphology and ovarian structure [62], but some analyses based on molecular data had led to incongruent results [63]. In addition, the phylogenetic position of Diplura was controversial [64]. Our phylogenetic estimate differed from that obtained by Luan et al. in their analysis of ribosomal RNA gene sequences. They inferred the relationships (Collembola + (Protura + Diplura)), with Diplura a monophyletic group, which was in accordance with our ML tree outgrouped by two Crustacean species (nodal support was relatively low in our analysis). Most other researches based on molecular data supported the monophyly of Diplura. Gao et al. [65] also retrieved a (Collembola + (Protura + Diplura)) clade using 18S and 28S rRNA gene sequences. But he stated that the nonstationarity of nucleotide frequencies among dipluran and proturan sequences because of the high content of GC, so their result might not be solid. Given the situation above, we suggested a more detailed research, including more dipluran taxa and more characters, would be necessary for a better understanding of the phylogenetic relationships of Diplura and of the subgroups within basal Hexapoda.

## Supporting Information

S1 Fig Phylogenetic relationships among basal Hexapods inferred from Neighbor-Joining analysis of 13 protein-coding gene sequences. 1 Protura species, 10 Collembola species and 3 Diplura species were used as outgroup.
(TIF)

S2 Fig Phylogenetic relationships among basal Hexapods inferred from Maximum likelihood analysis of 13 protein-coding gene sequences. 1 Protura species, 10 Collembola species and 3 Diplura species were used as outgroup.
(TIF)

S3 Fig Phylogenetic relationships among basal Hexapods inferred from Neighbor-Joining analysis of protein-coding gene sequences. 2 Crustacean species Capitulum mitella and Pollicipes mitella were used as outgroup.
(TIF)

S4 Fig Phylogenetic relationships among basal Hexapods inferred from Maximum likelihood analysis of protein-coding gene sequences. 2 Crustacean species Capitulum mitella and Pollicipes mitella were used as outgroup.
(TIF)

S1 Table List of universal primers used for PCR amplification. (DOCX)
