# Supplementary material for: The Complete Mitochondrial Genomes of Three Bristletails (Insecta: Archaeognatha): The Paraphyly of Machilidae and Insights into Archaeognathan Phylogeny
Source: PLoS One. 2015 Jan 30;10(1):e0117669. doi: 10.1371/journal.pone.0117669 (PMC4323385; doi:10.1371/journal.pone.0117669)
Supplement: S1 Table — (DOCX) [file pone.0117669.s005.docx]

| Table S1. List of universal primers used for PCR amplification. | | |
| --- | --- | --- |
|  |  |  |
| No. | Primer name | Nucleotide sequence (5’-3’) |
| 1 | N2-J-586 | CCTTTCCAYTTWTGATTYCC |
|  | TW-N-1284 | ACAGCTTTGAAGGCTATTAGTTT |
| 2 | TB-J-1350 | ACAAAACAAATAGCCTTCAA |
|  | N-2329 | ACTGTAAATATATGATGTGCT |
| 3 | J-3177 | ATAGAACAATTAATTTTYTTYCAYGATCA |
|  | N-3718 | GRTTTGYTCCACAAATTTCWGAGCATTG |
| 4 | CO3-J | ATAATTCAATGATGACGAGA |
|  | CO3-N | CCAATAAGAACATGTAGACC |
| 5 | N5-J7572 | AAAGGGAATYTGDGCTCTTTTMGT |
|  | N4-N8727 | AAGTCTTTAATTGCYTAYTCTTC |
| 6 | N4-J8641 | CCTCTMGAACATAANCCRTG |
|  | N4-N9153 | TGGGGTTATCAGCCWGARCG |
| 7 | J-11338 | CATATTCAGCCAGAATGATACTT |
|  | N-11845 | ACCACGATATCTTATGATA |
| 8 | J-12887 | CGGTTTGAACTCAGATCATGTAA |
|  | N-13398 | CACCTGTTTATCAAAAACA |
| 9 | 12S-J | TACTATGTTACGACTTAT |
|  | 12S-N | AAACTAGGATTAGATACCC |
| 10 | J-13331 | TGATTACGCTACCTTTGCACGGT |
|  | N-14588 | ATAATAGGGTATCTAATCCTAGTTT |
| Notes: M = A/C, Y = C/T, W = A/T, R = A/G, D=A/G/T, N=A/G/C/T. | | |
